# Molecular targets and signaling pathways regulated by interleukin (IL)-24 in mediating its antitumor activities

**DOI:** 10.1186/1750-2187-8-15

**Published:** 2013-12-30

**Authors:** Janani Panneerselvam, Anupama Munshi, Rajagopal Ramesh

**Affiliations:** 1 Department of Pathology, Stanton L Young Biomedical Research Center, The University of Oklahoma Health Sciences Center, Suite 1403, 975 NE 10th, Oklahoma City, OK 73104, USA; 2 Department of Radiation Oncology, University of Oklahoma Health Sciences Center, Oklahoma City, Oklahoma 73104, USA; 3 The Graduate Program in Biomedical Sciences, University of Oklahoma Health Sciences Center, Oklahoma City, Oklahoma 73104, USA; 4 Peggy and Charles Stephenson Cancer Center, University of Oklahoma Health Sciences Center, Oklahoma City, Oklahoma 73104, USA

**Keywords:** IL-24, Tumor suppressor, Cytokine, IL-10, Cancer, Apoptosis, Autophagy, Cancer stem cells, Clinical trial

## Abstract

Cancer remains a major health issue in the world and the effectiveness of current therapies is limited resulting in disease recurrence and resistance to therapy. Therefore to overcome disease recurrence and have improved treatment efficacy there is a continued effort to develop and test new anticancer drugs that are natural or synthetic - (conventional chemotherapeutics, small molecule inhibitors) and biologic (antibody, tumor suppressor genes, oligonucleotide) product. In parallel, efforts for identifying molecular targets and signaling pathways to which cancer cells are “addicted” are underway. By inhibiting critical signaling pathways that is crucial for cancer cell survival, it is expected that the cancer cells will undergo a withdrawal symptom akin to “de-addiction” resulting in cell death. Thus, the key for having an improved and greater control on tumor growth and metastasis is to develop a therapeutic that is able to kill tumor cells efficiently by modulating critical signaling pathways on which cancer cells rely for their survival.

Currently several small molecule inhibitors targeted towards unique molecular signaling pathways have been developed and tested in the clinic. Few of these inhibitors have shown efficacy while others have failed. Thus, targeting a single molecule or pathway may be insufficient to completely block cancer cell proliferation and survival. It is therefore important to identify and test an anticancer drug that can inhibit multiple signaling pathways in a cancer cell, control growth of both primary and metastatic tumors and is safe.

One biologic agent that has the characteristics of serving as a potent anticancer drug is interleukin (IL)-24. IL-24 suppresses multiple signaling pathways in a broad-spectrum of human cancer cells leading to tumor cell death, inhibition of tumor angiogenesis and metastasis. Additionally, combining IL-24 with other therapies demonstrated additive to synergistic antitumor activity. Clinical testing of IL-24 as a gene-based therapeutic for the treatment of solid tumors demonstrated that IL-24 is efficacious and is safe. The unique features of IL-24 support its further development as an anticancer drug for cancer treatment.

In this review we summarize the current understanding on the molecular targets and signaling pathways regulated by IL-24 in mediating its anticancer activity.

## Review

### Interleukin (IL)-24

The IL-24 gene originally referred to melanoma differentiation associated gene -7 (mda-7) belongs to the IL-10 cytokine superfamily. IL-24 DNA sequence includes an IL-10 signature and is composed of 7 exons and 6 introns and is located in a small 195 kb gene cluster on chromosome 1q31-32 [1,2]. Interestingly, several members of the IL-10 family of cytokines including IL-10, IL-19 and IL-20 are located on chromosome 1q31-32 [1,2]. Additional members of the IL-10 cytokine family located on different chromosome include IL-22, IL-26, IL-28A and IL-28B [3]. In this review we will refer mda-7 as IL-24 for consistency and interchange of IL-24 for mda-7 at any section of the review refers to the same gene and protein.

The IL-24 gene was originally discovered by subtraction hybridization method by exposing human melanoma cells (HO1 cell line) to the terminal differentiation inducing agents such as IFN-beta (IFN-β) and mezerin [4,5]. The cDNA of IL-24 is 1718-bp in length and encodes an evolutionarily conserved protein of 206 amino acids with a predicted molecular weight of 23.8 KD [5]. The 3′-untranslated region (UTR) of IL-24 mRNA has three consensus elements (AUUUA) and three polyadenylation signals (AAUAAA) which is involved in mRNA stability and regulation respectively [1,6]. Sequence analysis of IL-24 showed that it has an N-terminal hydrophobic signal peptide of 49 amino-acid in length that allows the IL-24 protein to be cleaved and secreted [7]. IL-24 has five phosphorylation (Serine 88, 101 & 161 and Threonine-111 &133) and three glycosylation sites (Cysteine 95, 109 and 126) [8,9]. Additionally, IL-24 protein has been shown to undergo ubiquitination and proteasome-mediated degradation [10]. IL-24 protein phosphorylation, glycosylation and ubiquitination suggest that the protein undergoes post-translational modification (PTM).

The IL-24 coding region has less than 19% amino acid homology with human IL-10 while the homology with other IL-10 family members varies between 15-40% [11,12]. The rat orthologue of human IL-24 is c49a/mob-5 which encodes a protein of 183 amino acids with a predicted molecular weight of 21.1-23 KD and has 63% homology with IL-24 [13-16]. FISP is the mouse orthologue of IL-24 and encodes a protein of 220 amino acids with the predicted mass of 25 KD and has 69% identity to human IL-24 at the protein level [17]. Although C49A/MOB-5 and FISP have significant homology with IL-24 protein, the biological function of these proteins is different from human IL-24. Among the several members of the IL-10 cytokine family, IL-24 is the only member that exhibits direct antitumor activity both *in vitro* and *in vivo*, the details of which will be discussed in the sections described below.

i) *Clinical correlation suggesting IL-24 is a tumor suppressor*. Clinical studies supporting IL-24 is a tumor suppressor or functions as a tumor suppressor was reported by two independent studies [18,19]. Immunohistochemical analysis of melanocytes, nevi and in different stages of melanoma showed IL-24 protein expression progressively decreased with disease progression from primary to metastatic phase with complete loss of expression in the metastatic phase [18,20]. Analysis of IL-24 expression in lung cancer showed an inverse correlation between IL-24 protein expression and disease progression [19]. Both of these studies showed loss of IL-24 protein expression correlated with disease progression and concluded IL-24 likely functions as a tumor suppressor. The studies also indicated that restoration of IL-24 protein expression might slow or suppress the disease.

ii) *Early preclinical study demonstrating IL-24 is a potential tumor suppressor*. The first preclinical report showing IL-24 is a tumor suppressor gene was demonstrated by Jiang et al. [6]. Molecular studies revealed both mRNA and protein for IL-24 was detectable in normal melanocytes. However, in melanoma tissues IL-24 mRNA but not the protein was detectable suggesting loss of IL-24 protein expression occurred during cellular transformation. Although the preclinical study preceded the clinical studies, the findings were in complete agreement with the clinical observation.

Follow-up studies showed that reintroducing exogenous IL-24 gene and restoring protein expression suppressed tumor growth both *in vitro* and *in vivo*[21]. Additionally, overexpression of IL-24 protein in normal cells did not elicit any cytotoxicity indicating IL-24 had selectivity towards tumor cells. These initial studies demonstrating IL-24 is a novel tumor suppressor/cytokine gene provided the impetus for conducting large scale studies testing IL-24 as an anticancer drug and unraveling the molecular mechanisms by which IL-24 exerted its antitumor activities.

iii) *IL-24 receptors*. Studies from two independent laboratories reported the identification of two receptors for IL-24 called IL-20 receptor (IL-20R) and IL-22 receptor (IL-22R) [15,22]. Both IL-20R and IL-22R exist as a heterodimer and is comprised of two subunits. IL-20R is comprised of IL-20R1 and R2 subunits while IL-22R is comprised of IL-22R1 and IL-20R2 subunits. Thus, IL-20R2 subunit is common and shared between IL-20 and IL-22 receptors. Within the IL-10 cytokine family, IL-19 can also bind to IL-20R while IL-20 can bind to both IL-20R and IL-22R [16,23]. Although there is substantial sharing of receptors among the members of the IL-10 cytokine family, the biological activities of IL-24, IL-19 and IL-20 are quite distinct with only IL-24 exhibiting receptor-mediated antitumor activity in human cancer cells. Thus, the ligand-receptor interaction is complex and reveals the existence of underlying differences in intracellular signaling upon ligand binding to the receptor. However, studies are yet to unravel the intracellular signaling triggered by IL-24 directing tumor cells to undergo cell death versus those mediated by IL-19 and IL-20 that do not trigger tumor cell death. In addition, distribution and expression of these receptor complexes in various tumor tissues and normal tissues has not been thoroughly investigated and is warranted.

Studies conducted by Parrish-Novak et al. showed that IL-24 protein binds with equal affinity to the two receptor complexes [24]. The binding of IL-24 to its receptors resulted in the activation of the signal transducer and activation of transcription-3 (STAT-3) and to a lesser extent STAT-1 [25]. Lower concentration of IL-24 protein was shown to activate STAT-3 whereas very high concentration of IL-24 activated STAT-1 [26]. Since STAT-3 is known to signal for cell survival and proliferation, the importance of IL-24 mediated STAT-3 activation was tested using STAT-3 inhibitors in IL-24 receptor-positive tumor cells [26]. Knock-down of STAT-3 did not abrogate IL-24 protein-mediated cytotoxicity indicating STAT-3 was not required for IL-24-mediated antitumor activity. This study also demonstrated that IL-24 protein selectively kills receptor-positive but not receptor-negative tumor cells when bound to its receptor thus providing an extracellular-protein mediated tumor cell killing. No cytotoxicity was observed when IL-24 protein bound to receptor-positive normal cells.

### IL-24-mediated antitumor activities involve regulation of multiple signaling pathways

#### a) Tumor cell killing

Since the initial report of IL-24 functioning as a tumor suppressor gene, studies from our laboratory and others have tested IL-24 as an anticancer drug for the treatment of a broad-spectrum of human cancers [11,12,26-28]. The consensus from the large number of studies reported till date is that IL-24 functions as a tumor suppressor and IL-24-mediated cytotoxicity is selective towards tumor cells with minimal to no toxicity to normal cells. Another observation that is in agreement with all of the current reports is that the cellular signaling pathways that are regulated by IL-24 varies in different cancer cells tested and is cell-type dependent. However, all of the signaling pathways, irrespective of the cancer cell type, converge downstream on cellular apoptosis resulting in caspase activation and tumor cell death.

The various molecular signaling pathways that are regulated by IL-24 in human cancer cells leading to tumor cell killing is discussed below.

##### Apoptosis

Initial studies conducted in our laboratory showed adenovirus (Ad)-mediated IL-24 gene delivery in human lung cancer cells resulted in induction of tumor cell apoptosis also commonly referred to type-I programmed cell death (PCD) [29]. Molecular studies revealed Ad-IL24 activated the intrinsic apoptotic pathway as determined by the activation of cytochrome-C, caspase-9 and -3 [29]. Follow-up studies demonstrated IL-24 mediated tumor cell apoptosis was independent of the mutational status for p53, Ras, Bax and Rb [30]. Activation of c-Jun NH2-terminal kinase (JNK) by IL-24 and its requirement in IL-24-mediated apoptotic cell killing when combined with radiation was first demonstrated in human A549 lung cancer cells [30]. Follow-up studies in prostate cancer and glioma showed JNK activation when IL-24 treatment was combined with radiation [31,32]. Subsequent to JNK activation, modulation of pro- (Bax and Bak) and anti-apoptotic (Bcl-Xl and Bcl-2) proteins were demonstrated in the prostate cancer model. Treatment with SP600125, a JNK inhibitor abrogated the tumor cell killing demonstrating JNK was required for IL-24-mediated tumor cell killing [32].

In melanoma cells, p38MAPK signaling was demonstrated to be important for IL-24-mediated tumor cell killing [33]. Activation of p38MAPK by IL-24 led to marked induction of DNA-damage-inducible (GADD) family of genes that included GADD153, GADD45α and GADD34 that culminated in apoptosis. A modest induction of GADD45γ was also noted in the study. Inhibiting p38MAPK with SB203580, a specific small molecule inhibitor abrogated IL-24-induced melanoma apoptosis [33]. Knock-down of GADD family of genes using antisense oligonucleotides also abolished IL-24- mediated cell death. Induction of GADD family of genes was also demonstrated in Ad-IL24-treated glioblastoma multiforme, prostate cancer, breast cancer and in pancreatic cancer cells that resulted in tumor cell apoptosis [34].

While reports of IL-24 functioning as a tumor suppressor and its ability to regulate apoptotic signaling pathways were exploding, Pataer et al. showed Ad-IL24 treatment of lung cancer cells resulted in activation of double stranded RNA-dependent protein kinase (PKR) protein resulting in tumor cell killing [35]. PKR belongs to the eukaryotic initiation factor (eIF) 2α kinase family. Additionally, in the same study they showed that activation of PKR by IL-24 resulted in the phosphorylation of eIF2α, TYK2, Stat1, Stat3 and p38MAPK proteins all of which are known to play a role in protein synthesis, growth suppression and induction of apoptosis. Follow-up studies by the same group investigated whether protein-protein interaction between IL-24 and PKR was important for tumor cell killing. Their study unveiled a new observation showing IL-24 physically bound the double stranded RNA-dependent protein kinase (PKR) protein and its binding resulted in phosphorylation of both proteins [36] that contributed to tumor cell killing. Additionally, requirement of PKR for IL-24-mediated cell killing was demonstrated using PKR wild-type (+/+) and PKR null (**-/-**) mouse embryo fibroblast (MEF) cells. Ad-IL24 exhibited cytotoxicity towards PKR+/+ but not to PKR -/- MEFs. These results established tumor suppressor activity of IL-24 required PKR. Follow-up studies in our laboratory tested the requirement of PKR in other cancer cell lines. Ad-IL24 treatment of prostate cancer cells showed PKR activation in LNCaP cells but not in DU145 cells [37]. In ovarian cancer cells, activation of Fas-Fas ligand pathway was shown to be more important in IL-24-mediated cell death than the PKR activation [38]. It is thus evident that the requirement of PKR in IL-24-mediated cell death is cell-type dependent and that IL-24 can exert its antitumor activity independent of PKR.

Another mechanism related to PKR is the activation of PKR like endoplasmic reticulum kinase (PERK) by IL-24 [39,40]. PERK like PKR belongs to the eIF-2α family. Treatment of cells with Ad-IL24 resulted in IL-24 protein expression that bound and inactivated HSP70 family chaperone BiP/GRP78, which in turn promoted dissociation and activation of PKR-like endoplasmic reticulum kinase (PERK) resulting in initiation of tumor cell apoptosis. Involvement of PERK has also been demonstrated in IL-24-mediated apoptosis that involves induction of reactive oxygen species (ROS). Exogenous expression of IL-24 in tumor cells resulted in ROS production which in turn deregulated mitochondrial function via PERK dependent generation of lipid second messenger ceramide leading to cell death [12,31,41,42]. Increase in intracellular ceramide levels facilitated calcium ion dependent generation of ROS production that amplified the autocrine signaling loop and effected tumor cells in both autocrine and paracrine fashion culminating in cell death.

Li et al. recently demonstrated IL-24 increases the level of ROS, followed by the induction of differentiation and programmed cell death, in SH-SY5Y neuroblastoma cells [43]. Subsequent studies conducted in prostate cancer cells showed treatment with antioxidants such as N-acetyl-L-cysteine and Tiron or with inhibitors of mitochondrial permeability transition (cyclosporine A and bongkrekic acid) abrogated Ad-IL24-induced apoptosis [44]. In contrast, treatment with agents that induce ROS production (arsenic trioxide, NSC656240 and PK11195) enhanced Ad-IL24 induced cellular apoptosis. Ad-IL-24 when combined with a ROS inducing agent demonstrated enhanced antitumor activity in a pancreatic mouse model and was independent of the K-ras status [45]. Ectopic expression of Bcl-2 and Bcl-XL inhibited Ad-IL24 induced mitochondrial changes, ROS production and apoptosis, which further substantiates the involvement of mitochondrial dysfunction in Ad-IL24 induced apoptosis [41]. In contrast to these findings, Lee et al. reported IL-24 inhibited hydrogen peroxide (H_2_O_2_) induced ROS production in normal vascular smooth muscle cells (VSMC) by reducing mitochondrial H_2_O_2_ production and by enhancing the expression of antioxidant enzymes [46]. It is evident from these reports that IL-24 selectively induces ROS-mediated cell death in tumor cells but not in normal cells.

Negative regulation of β-catenin and phosphatidylinositol 3-kinase (PI3K) pathways is another mechanism by which IL-24 exerts its anticancer activity in human breast, lung and pancreatic cancer cells [26,47]. In continuation with these reports our laboratory has pursued to dissect the PI3K/Akt/mTOR pathway in human lung cancer cells that have been stably transfected to express exogenous IL-24. Preliminary results indicate IL-24 effectively inhibits Akt1/2 and its downstream target mTOR in lung cancer cells resulting in inhibition of cell growth, cell migration and invasion [48].

From the above reports it is evident that IL-24 induces tumor cell apoptosis by modulating various signaling pathways that is cell-type dependent.

##### Autophagy

Autophagy or type-II PCD occurs under physiological and pathological conditions in response to cellular stress such as nutrition deprivation, inflammation, hypoxia, and exposure to various drug treatments. Although autophagy was originally defined as a cell survival mechanism by which cells and cellular organelles are degraded and cleared without activating the host immune system. However, studies have demonstrated autophagy also plays an important role in cancer cell survival and death [49]. While there is fair amount of literature supporting cancer cells utilize the autophagy pathway for their survival, there also exists a significant number of reports demonstrating exposure of tumor cells to anti-cancer drugs results in autophagy-mediated tumor cell death [50]. Thus, autophagy plays a role in both cell survival and cell death and the switch from survival to death likely depends on the cellular stress threshold. On the basis of these observations, several laboratories are attempting to manipulate the autophagic process in cancer cells as a new method of cancer therapy.

Interests in studying whether IL-24 regulates autophagy in cancer cells arises from the initial observation and reports made by our laboratory and others [51,52]. We and others showed enforced expression of IL-24 in tumor cells resulted in accumulation of IL-24 protein in the endoplasmic reticulum (ER) that lead to activation of the unfolded protein response (UPR) and expression of molecular chaperones such as glucose-regulated protein (GRP) 78/immunoglobulin binding protein (BiP) [53,54]. Additionally, expression of PERK and activating transcription factor (ATF)-4 which are normally bound to and inactivated by BiP/GRP78 was shown to be regulated by IL-24. Activation of the UPR/GRP78/BiP pathway restores proper protein folding and thus reduces ER stress and prevents cells from undergoing cell death. However, accumulating data in the recent years suggests that autophagy is also initiated in response to ER stress caused by an overload of misfolded proteins [55].

Since IL-24 induced ER stress and regulated the UPR/GRP78/BiP pathway, the possibility of IL-24 inducing autophagy-mediated tumor cell death was investigated. Treatment of glioma cells with glutathione-S-transferase (GST)-IL24 fusion protein resulted in simultaneous activation of both autophagy and apoptosis [40]. Park et al. showed GST-IL24 protein-mediated autophagy in glioma cells was dependent on PERK-mediated ER stress that involved inactivation of ERK1/2 and activation of the JNK pathway [56,57]. In the same study the authors showed GST-IL-24 induced PERK-dependent vacuolization of LC3-expressing endosomes formation in glioma cells that was suppressed when treated with inhibitors of autophagy. Finally, autophagy was shown to overlap with activation of the pro-apoptotic pathway culminating in tumor cell death. Yacoub et al. showed treatment of glioma cells with adenovirus (Ad)-IL24 induced ER stress and triggered intracellular ceramide production and ROS generation resulting in autophagic cell death [58]. Ad-IL24 when combined with OSU-03012, an autophagy inducing drug, enhanced the antitumor activity in glioma cells by increasing ER stress and simultaneously reducing anti-apoptotic (MCL-1 and BCL-XL) protein expression [59]. In renal cell carcinoma, IL-24 when combined with histone deacetylase inhibitors (HDACIs) elevated intracellular Ca2+ level and increased ROS production resulting in autophagy and cell death [60]. In prostate cancer cells but not in normal prostate epithelial cells, IL-24 induced autophagy through a canonical signaling pathway involving beclin-1, AuTophaGy-related (ATG)-5 and hVps34 [61]. Autophagy was observed to occur at earlier time points (< 24 h) that switched to apoptosis by 48 h after IL-24 treatment. Concurring with these findings, Yokoyama et al. showed human melanoma cells when treated with IL-24 protein induced beclin-1 resulting in autophagy at 24 h after treatment [62]. However, time course studies revealed switching from autophagy to apoptosis (unpublished data).

In contrast to the studies described above demonstrating IL-24 induced autophagy facilitated cell killing, Yang et al. using a conditionally replicating adenovirus (ZD55) reported exogenous expression of IL-24 in chronic lymphocytic leukemia B-cells induced autophagy via upregulation of beclin-1 that promoted cell survival [63]. However, when the cells were treated with wortmanin, an autophagy inhibitor, IL-24-mediated autophagosomes were inhibited resulting in killing of the leukemia cells.

It is evident from the above reports that IL-24-mediated cell killing involves both autophagy and apoptosis. The study results also suggest that combining IL-24 with activators of apoptosis and autophagy will produce enhanced antitumor activity and will be beneficial in cancer treatment. However, caution needs to be taken when IL-24-based combination therapy are planned and should be tailored based on the cancer type being studied. As evident from the leukemia study, inhibiting autophagy will be beneficial for producing enhanced antitumor activity with IL-24.

#### b) Bystander effect

Initial studies conducted in our laboratory and others focused on testing IL-24 as a cancer gene therapeutic using viral and non-viral vectors and investigating the molecular mechanism of cell killing. However, since IL-24 DNA sequence revealed a secretory signal sequence it was postulated that IL-24 protein is secreted. Studies from our laboratory and others have demonstrated IL-24 protein is glycosylated and secreted [2,64]. The question that arose next was whether the secreted protein had any antitumor activity and if IL-24 receptors were required for the activity? Another question raised was whether the secreted IL-24 protein had any inhibitory effect on neighboring tumor cells that did not express IL-24? Finally, whether IL-24 exerted its antitumor activity by both intracellular and extracellular mechanism was to be resolved.

The answers to the questions were partly resolved by studies conducted in our laboratory and others. Treatment of human pancreatic tumor cells and melanoma cells with human IL-24 protein produced in eukaryotic cells exhibited potent cytotoxicity [11,26]. The authors in these studies showed the cytotoxicity was selective towards receptor-positive tumor cells and spared receptor-positive normal cells. Additionally, it was demonstrated that IL-24 protein utilized its receptors for mediating the antitumor activity in tumor cells. Receptor-negative tumor cells did not undergo cell death when treated with IL-24 protein. Although ligand-receptor mediated cell killing was demonstrated, the receptor utilization by IL-24 protein varied among tumor types. For example in melanoma cell lines, IL-20 receptor was shown to be required while IL-22 receptor was required in pancreatic cancer cell lines. Thus, the usage of the receptors can vary among tumor cell lines [11,26]. Molecular studies revealed IL-24 on binding to its receptors resulted in activation of signal transducer and activator of transcription (STAT)-3 and expression of the pro-apoptotic Bax protein resulting in apoptotic cell death [26]. However, knock-down of STAT-3 did not abrogate IL-24 protein-mediated killing of the receptor-positive tumor cells indicating STAT-3 activation was not required for tumor cell killing. Concurring with these findings was the report showing IL-24 protein produced in bacteria killed human breast and prostate cancer cells with no toxicity to normal cells [12]. The results from all of the studies revealed that glycosylation of IL-24 protein was not required for mediating its antitumor activity but was required for its stability in the extracellular environment.

Subsequent studies showed receptor-positive tumor cells transduced with Ad-IL-24 or transfected with IL-24 plasmid DNA and overexpressing IL-24 when mixed with receptor-negative tumor cells resulted in killing of both receptor-positive and –negative cells [29,65]. This observation indicated IL-24 could exert a “bystander” tumor killing effect. Su et al. demonstrated normal cells infected with Ad.MDA-7 and co-cultured with cancer cells resulted in reduced cell viability [64]. When the normal and tumor cells were separated by agar overlay thereby eliminating any physical contact between the two cell types, tumor cell killing still occurred. These studies elegantly demonstrated that IL-24 protein could diffuse through agar, bind to receptor-positive tumor cells and reduce tumor cell viability, alter anchorage-independent growth, and radiosensitize culminating in apoptotic cell death.

*In vivo* studies demonstrated mice implanted with a mixture of IL-24 producing human embryonic kidney cells (HEK)-293 and human receptor-negative A549 lung tumor cells underwent tumor growth inhibition [66]. Molecular studies revealed IL-24 protein was secreted and circulating IL-24 protein was detectable in serum collected from mice. The antitumor activity was proven to occur by IL-24 protein affecting the IL-24 receptor-positive tumor endothelial cells and inhibiting tumor angiogenesis. Additionally, inhibition of contralateral tumors was demonstrated establishing the concept of IL-24 protein can suppress tumor growth by exerting a “bystander effect”.

In another study, intratumoral injection of Ad-IL24 into a flank tumor resulted in shrinkage of contralateral tumor [67]. In this study, human T47D breast carcinoma cells were implanted into both flanks of nude mice and Ad.MDA-7 was injected only in the left side of the tumor. Apart from having a significant reduction in the tumor growth treated with Ad.MDA-7, inhibition of the contralateral tumor that was not treated with Ad.MDA-7 was observed demonstrating the ‘bystander’ tumor killing activity for IL-24 protein. Additional studies have confirmed IL-24 protein-mediated tumor cell killing [68-70]. Apart from direct tumor cell killing and inhibiting tumor angiogenesis, additional molecular mechanism contributing to the bystander effect has been the activation of the host immune system, induction of ER stress and generation of ROS [71,72]. Finally, occurrence of bystander effect in humans diagnosed with cancer and treated with Ad-IL24 was demonstrated in a Phase I clinical trial [73,74]. Results from the clinical trial showed intratumoral administration of Ad-IL24 (INGN 241) resulted in tumor cell apoptosis both at the treated site and in tumor cells at a distant site.

The results from all of these studies clearly established secreted and circulating IL-24 protein exhibited “bystander effect” both *in vitro* and *in vivo*. This unique feature provides the opportunity in using IL-24 both as a gene- and protein-based therapeutic. Additionally, testing IL-24 as a gene therapeutic overcomes the limitations of vector transduction efficiency and does not require the tumor in its entirety to be transfected and express IL-24 for producing observable antitumor activity.

#### c) Metastasis

The rationale to test IL-24 for its anti-metastatic activity arose from the clinical observation made by Ellerhorst et al. who showed inverse correlation between IL-24 expression, tumor cell invasiveness and disease progression in melanoma [20]. The inhibitory activity of IL-24 on tumor cell metastasis and invasion was first demonstrated by our laboratory using lung cancer as a model [75]. *In vitro* studies demonstrated Ad-IL24 markedly reduced the cell invasion and migration ability of human H1299 and A549 lung cancer cells [75]. Molecular studies revealed IL-24 inhibited PI3K/Akt, matrix metalloproteinase (MMP)-2 and -9, and focal adhesion kinase (FAK) protein expression. All of these proteins have previously been shown to play a role in tumor cell survival and metastasis [76-78]. Additional studies from our laboratory showed IL-24 enhanced E-Cadherin expression, a protein that plays a role in cell-cell contact and adhesion [26]. The inhibitory activity of IL-24 on cell migration and invasion was shown to be independent of tumor cell killing. *In vivo*, treatment of lung tumor-bearing mice with a cationic lipid-based nanoparticle containing IL-24 plasmid DNA reduced lung metastasis [79]. Follow-up studies from our collaborators laboratory showed IL-24 inhibited the PI3K and Wnt/beta-catenin signaling pathway in breast cancer cells [26]. Both, PI3K-AKT-mTOR pathway and Wnt signaling pathway have been shown to be involved in tumor cell invasion and metastasis [26,80]. Concurring with our study results were the reports from other laboratories demonstrating IL-24 exhibited antimetastatic activity in pancreatic cancer cells [76-78]. All of these findings provide evidence that IL-24 inhibits multiple signaling pathways that are associated with tumor cell metastasis.

More recent studies conducted in our laboratory has revealed IL-24 when expressed at pharmacological levels in human H1299 lung cancer cells inhibited the Akt/mTOR signaling pathway resulting in suppression of the tumor cell migratory function [48]. Expression of myristoylated Akt protein resulted in diminished IL-24 inhibitory activity on cell migration and invasion demonstrating IL-24-mediated anti-metastatic activity occurred by targeting Akt (unpublished data). Investigation into molecular signaling upstream of Akt has revealed IL-24 suppresses the chemokine CXCR4/CXCL12 axis (unpublished data). CXCR4 is upstream of Akt and activation of the CXCR4/CXCL12 results in signal transduction downstream that involves the Akt/mTOR pathway. Studies have demonstrated CXCR4/CXCL-12 signaling is important for cell migration and invasion and contributes to tumor metastasis [81-85]. In fact, inhibitors of CXCR4 are currently in clinical testing for lung cancer and other solid tumors. On the basis of our preliminary findings we speculate that IL-24-mediated antimetastatic activity primarily occurs by inhibiting the CXCR4/CXCL12 pathway. Additional preclinical studies are currently being conducted in the laboratory to define how IL-24 regulates CXCR4/CXCL12 and identify the intermediary signaling proteins involved in the cross-talk between CXCR4 and Akt and that might also be modulated by IL-24.

From a clinical perspective, the advantage of using IL-24 as an anticancer drug is that tumor cells often use multiple signaling pathways to escape from the cytotoxic effects of a drug. Additionally, inhibiting one pathway in cancer cells often results in the activation of a redundant or alternate pathway thus resulting in cell survival. Therefore a cocktail therapy would be required. These potential problems are minimized using IL-24 as a therapeutic at least in the preclinical studies where IL-24 inhibited multiple pathways resulting in cell killing and inhibition of metastasis.

#### d) Angiogenesis

Angiogenesis is a complex process, which involves series of molecular events and signaling cascade including formation of new blood vessels, endothelial cell proliferation, disruption of existing extracellular matrix and formation of new matrix. Angiogenesis is a requirement for normal physiologic conditions. However, under pathological conditions such as in cancer, angiogenesis plays an important role in contributing to tumor growth and development of metastasis [86]. Thus, the concept of antiangiogenic therapy was developed with the hypothesis “inhibiting tumor angiogenesis would deprive tumor cells of nutrients and oxygen” resulting in collapse of blood supply to the tumors resulting in tumor cell death and shrinkage [87,88]. On the basis of this concept several antiangiogenic agents have been developed and tested with few showing promise in the clinic [89-98]. Thus there is a continuum to develop and test new and improved antiangiogenic agents of biological and synthetic origin.

The concept of testing IL-24 for its antiangiogenic activity arose from a serendipitous observation made in our laboratory. Treatment of lung tumor xenograft with Ad-IL24 resulted in tumor growth inhibition that was associated with reduction in number of CD31 positive endothelial cells, a marker indicative of reduced blood vessels in the tumor. This initial observation lead us to ask the question of whether IL-24 inhibited tumor vascularization and the underlying molecular mechanism? *In vivo* chamber window studies showed Ad-IL24 significantly inhibited neo-angiogenesis [99]. Follow-up studies focused on demonstrating IL-24 protein possessed antiangiogenic activity. Addition of human IL-24 protein to human umbilical vein endothelial cells (HUVEC) and human lung microvascular endothelial cells (HMVEC-L) resulted in a dose-dependent inhibition of endothelial cell differentiation (ECD) but not endothelial cell proliferation [66]. Additionally, IL-24 potently inhibited vascular endothelial growth factor- (VEGF) and basic fibroblast growth factor- (bFGF) induced endothelial cell migration. The IL-24 inhibitory effect on HUVEC and HMVEC was 10–50 times more potent than the inhibitory effects induced by interferon (IFN) –γ, endostatin, and inducible protein (IP)-10 [66]. The inhibitory effect of IL-24 was shown to occur via the IL-22 receptor and a requirement for IL-22R1 subunit expression on endothelial cells was demonstrated. The intracellular signaling that occurs following IL-24 binding to its receptor on endothelial cells remains unknown. This is an area that needs to be investigated in detail for improving our understanding of IL-24 biology and angiogenesis. It is possible that IL-24 inhibits the Akt/mTOR signaling pathway in endothelial cells akin to that observed in the tumor cells. This possibility is likely to occur since in a separate study we showed tissue culture supernatant collected from Ad-IL24-infected tumor cells and rich in secreted IL-24 protein when added to HUVEC cells showed reduced activation of Akt [47,75]. However, additional mechanistic studies are warranted to dissect the signaling pathway that triggers the inhibitory activity on endothelial cells.

*In vivo* studies confirmed the *in vitro* findings and demonstrated IL-24 protein suppressed growth of lung tumor xenograft by inhibiting tumor angiogenesis [75]. *In vivo* studies showed human embryonic kidney 293 cells (HEK 293) stably transfected and expressing IL-24 (HEK293-IL24) when mixed with human A549 lung tumor cells and implanted into nude mice resulted in suppression of tumor growth when compared to tumor growth in mice implanted with mixture of unmodified parental HEK293 and A549 cells [66]. In addition to tumor growth inhibition, apoptosis of tumor endothelial cells and tumor cells, reduced number of CD31 positive endothelial cells and decreased hemoglobin level were also evident [66]. These findings demonstrated that IL-24 protein directly inhibits tumor angiogenesis and possess antiangiogenic activity. Additionally, IL-24 protein secreted from HEK293-IL24 cells and circulating in serum exhibited “bystander effect” on tumors implanted at a distant site in the mice. In this experiment, A549 lung tumors were implanted in the lower right flank of the nude mice, when the tumors reached the size of 50–100 mm3, HEK293 or HEK293-IL24 cells were implanted subcutaneously in the upper right flank and tumor growth were monitored. A significant delay in tumor growth (40-50% reduction) was observed on both flanks of mice that were implanted with a mixture of tumor cells and HEK 293-IL24. Further, the animals implanted with HEK293-IL24 did not show any toxicity indicating that circulating IL-24 protein had no non-specific toxicity towards normal tissues. Our findings showed that IL-24 protein in blood circulation can produce systemic and direct anticancer activity *in vivo.*

In a separate study we showed IL-24 can also modulate angiogenesis by suppressing growth factors produced by tumor cells. Our study showed treatment of lung tumor cells with Ad-IL24 resulted in diminished expression of VEGF, IL-8, FGF and transforming growth factor (TGF) mRNA in lung tumor cells [99]. Follow-up study by Nishikawa et al. showed Ad-IL24 inhibited VEGF, bFGF, and IL-8 protein expression in human lung tumor xenografts [100]. Furthermore, combining radiation therapy with Ad-IL24 resulted in greater inhibition of VEGF, bFGF, IL-8 and tumor neovascularization resulting in enhanced antitumor activity. The results from this study concurred with our own findings and suggested IL-24 could also indirectly inhibit tumor angiogenesis by reducing growth factor expression by tumor cells.

Follow-up studies in our laboratory demonstrated IL-24 inhibited VEGF expression in lung and prostate cancer cells through the Src pathway [101]. IL-24 was shown to directly inhibit Src kinase activation resulting in reduced transcription and translation of VEGF. Moreover, conditioned tissue culture supernatant obtained from Ad-IL24 treated tumor cells when added to actively growing HUVEC cells markedly diminished VEGF receptor 2 (VEGFR2)-mediated AKT signaling that resulted in induction of endothelial cell growth arrest and apoptosis [101]. However, these inhibitory effects on HUVEC cells were abrogated on exogenous addition of recombinant VEGF protein to the tissue culture media obtained from Ad-IL24-treated tumor cells. The results from this study showed IL-24 inhibited VEGF expression in tumor cells that resulted in diminished signaling to endothelial cell survival thereby causing an antiangiogenic effect. To further substantiate the findings, we combined Ad-IL24 with the clinically approved antiangiogenic drug, Bevacizumab (Avastin). Bevacizumab is an anti-VEGF antibody that binds and prevents secreted VEGF from binding to its receptor, VEGFR2. *In vitro* and *in vivo* studies demonstrated enhanced antitumor activity when Ad-IL24 was combined with Bevacizumab [101]. Molecular studies showed both Ad-IL24 and Bevacizumab treatment reduced VEGF expression levels in tumor cells both *in vitro* and *in vivo*. However, combination therapy showed the greatest reduction in VEGF expression. Thus, IL-24 suppresses angiogenesis by inhibiting the expression of growth factors produced by tumor cells.

Results from all of the studies described above clearly establish IL-24 exerts its antiangiogenic activity by direct and indirect mechanism of action on tumor endothelial cells and tumor cells respectively. Although we demonstrated IL-24 activity on VEGF, it is still not known how other growth factors are modulated by IL-24. Additional information obtained by conducting more detailed studies will allow testing of new combination therapies with novel antiangiogenic agents.

##### Combination therapy

It will be evident from the detailed description provided in the earlier sections that IL-24 is a potent tumor suppressor/cytokine that modulates several signaling pathways that are required for tumor cell survival, metastasis and angiogenesis. Given the plethora of information on IL-24, several laboratories including our own laboratory have tested IL-24-based combination therapies. Treatment of lung tumor cells with Ad-IL24 in combination with radiation downregulated DNA-repair enzymes resulting in enhanced radiation-induced DNA damage in tumor cells and tumor regression [100]. Treatment of human breast cancer xenograft with Ad-IL24 and radiation produced complete tumor regression [102]. Zhao et al. showed the tumor suppressive effect of dual gene therapy (ING4-inhibitor of growth family member 4 and IL-24) combined with radiotherapy in the breast cancer cells [103]. In this study Ad. ING4/IL-24 gene therapy and radiotherapy suppressed cell proliferation and induced apoptosis in breast cancer cells. In another study, Ad-IL24 when combined with cyclooxygenase (COX)-2 inhibitor produced enhanced radiosensitization of breast cancer cells [104]. These findings reveal the advantage and clinical relevance of combining IL-24-based gene therapy with radiotherapy.

Ad-IL24 when combined with non-steroidal anti-inflammatory drug (NSAIDS) such as sulindac suppressed nuclear factor (NF) kappa B resulting in enhanced antitumor activity [105]. Subsequent studies from other laboratories concurred with our findings and demonstrated enhanced antitumor activity when combined with other NSAIDS [45]. Enhanced antitumor activity has also been demonstrated when Ad-IL24 was combined with molecularly targeted antisense oligonucleotides, biologic therapy, antioxidants and other novel agents in various types of cancer cells [106-110]. McKenzie et al. showed cytotoxic effects of Ad-IL24 against breast cancer cells was enhanced when combined with Trastuzumab (Herceptin) [111]. Combination of Ad-IL24 and human tumor necrosis factor-alpha (rhTNF) also showed synergistic therapeutic effect in human prostate cancer cells [112]. Similarly, treatment of melanoma cells with Ad-IL24 plus Temozolomide produced greater antitumor activity [113].

Since these early reports showing combining IL-24 with other therapies produced greater antitumor activity, a slew of studies have recently been conducted reporting similar findings. For example, combination of oncolytic adenovirus expressing IL-24 with chemotherapeutic agents dramatically enhanced the cytotoxic effects through induction of apoptosis against cancer of the breast, colon, liver, lung, brain, pancreas, and melanoma [110,114-123]. In yet another study, treatment of hepatocellular carcinoma (HCC) cells *in vitro* with IFN-alpha in combination with an oncolytic adenovirus expressing IL-24 (SG600-IL-24) resulted in tumor cell apoptosis. Molecular studies showed increase in STAT-1 and SOCS1 protein expression while decrease in the expression of metastatic and angiogenic proteins such as MMP-2, XIAP, OPN and VEGF [124]. *In vivo* studies showed IFN-alpha plus SG600-IL-24 treatment resulted in significant reduction in tumor growth and increased the survival of mice. All of these studies provide evidence that combining IL-24-based therapy with other therapies will be beneficial and produce maximal antitumor activity with minimal to no toxicity to normal tissues. Combining IL-24 with other treatments will also reduce the treatment doses required thereby circumventing dose-limited toxicity and potentially avoid or delay development of therapy resistance.

##### Cancer stem cells

**Figure 1:**
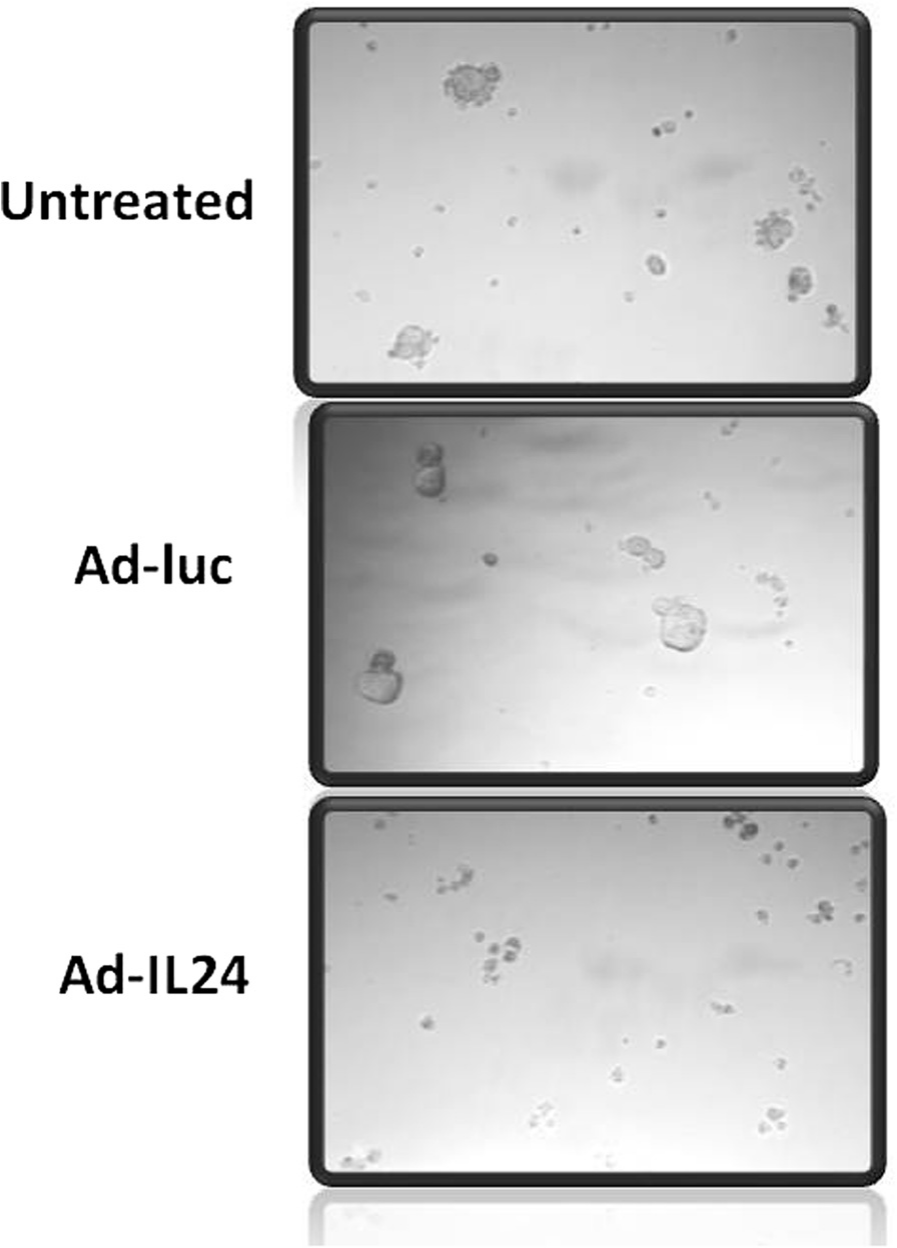
**IL-24 suppresses tumorosphere formation by lung cancer stem cells.** Aldehyde dehydrogenase positive (ALDH^+^) human lung cancer stem cells (CSCs) when treated with Ad-IL24 showed reduced tumorosphere formation *in vitro*.

The last decade has witnessed several laboratories including our own testing IL-24 as anticancer drug against established *in vitro* and *in vivo* tumor models. With the discovery of cancer stem cells (CSCs) or tumor-initiating cells (TICs) and their role in contributing to tumor relapse and therapy resistance [125], emphasis has recently shifted towards testing the antitumor activity of IL-24 on CSCs. A recent study reported CD44^+^/CD24^-/low^ breast CSCs were susceptible to Ad-IL24 treatment both *in vitro* and *in vivo*[126]. No toxicity was observed towards normal stem cells. Additional observations made in this study was Ad-IL24 exhibited “bystander therapeutic effect” on contralateral untreated CSC-induced tumors in mice. Mechanism of CSC death was shown to occur via ER stress-mediated apoptosis as evidenced by the upregulation of BiP/GRP78, GRP94 and GADD153 proteins and activation of eIF2α protein [126]. IL-24 has also been reported to efficiently reduce the proliferation and induce apoptosis in myeloid leukemia cells and in leukemia stem like cells [127,128]. More recently, studies undertaken in our laboratory showed Ad-IL24 treatment of aldehyde dehydrogenase positive (ALDH^+^) human lung CSCs reduced the number of tumorospheres (unpublished data; Figure 1). The results from these studies demonstrate IL-24 can efficiently eliminate both tumor cells and CSCs and thus IL-24-based therapy is an attractive therapy option for the treatment of cancer.

##### IL-24 mediated cellular signaling - clinical observation

Based on the extensive preclinical data demonstrating the antitumor activities of IL-24, a Phase I clinical trial was initiated to test the maximum tolerated dose (MTD) and toxicity of Ad-IL24 treatment in cancer patients. Secondary end points included biological measurements of transgene expression, molecular markers and tumor cell death. Study results at the end of trial revealed Ad-IL24 therapy was safe and well-tolerated with no untoward treatment-related toxicity observed [73,74]. Biological assays showed 10% to 30% of the tumor mass was transduced following Ad-IL24 treatment that resulted in 70% of the tumor cells showing signs of apoptosis. Additionally, expression of exogenous IL-24 protein was observed in areas of tumor tissues that were outside and beyond Ad-IL24 injection site suggesting IL-24 protein was secreted that diffused throughout the tumor. These findings support the occurrence of IL-24-mediated bystander antitumor activity, an observation that concurred with preclinical study results [11,26,66]. Additionally, increase in tumor necrosis factor-α (TNF-α), IL-6, IL-10, and interferon-γ (IFN-γ) cytokine levels and CD8 positive-T cells were demonstrated in patients treated with Ad-IL24 suggesting activation of the pro-immune response, another observation that was in agreement with preclinical data [72,73]. Thus, the existence of a strong correlation between preclinical and clinical study results warrant further testing of IL-24-based cancer therapy either as monotherapy or in combination with other therapeutics in the clinic for treatment of human cancers.

## Conclusions and future directions

**Figure 2:**
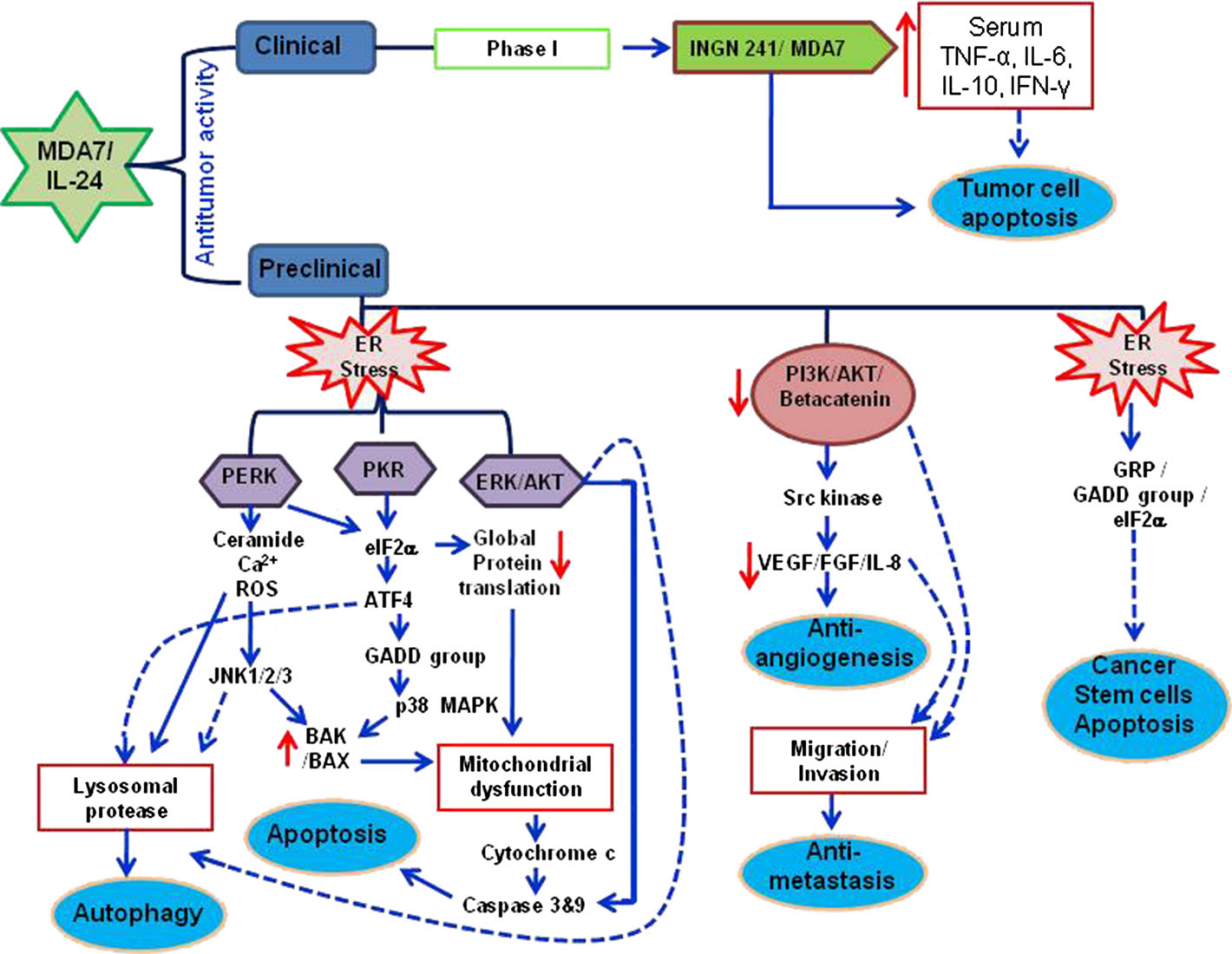
**Molecular signaling pathways regulated by MDA-7/IL-24 in human cancer cells.** Schematic summarizes the findings made in clinical and preclinical studies demonstrating molecular signaling pathways that are regulated by IL-24 thereby inhibiting cancer cell growth, invasion and metastasis.

Results from preclinical and clinical studies have established IL-24, a member of the IL-10 super-family, functions as a tumor suppressor/cytokine gene. IL-24 is the only IL-10 family member that exhibits antitumor activity thus separating itself from other family members. Although IL-24 is not a classical tumor suppressor, its ability to inhibit multiple cell signaling pathways that are required for tumor growth, invasion, metastasis and angiogenesis places it in a unique class of anticancer agents (Figure 2). It is also evident that combining with different therapies results in enhanced anticancer activity providing an opportunity for developing IL-24-based personalized therapy for treating patients diagnosed with various types of cancer. Finally, the ability to kill CSCs will likely reduce disease relapse and improve overall five-year survival of cancer patients. Added advantage that is foreseen is that IL-24 therapy will improve the patient’s quality of life as no adverse events or treatment-related toxicity was observed in the clinical trial. Although use of IL-24 as an anticancer drug for treatment of cancer is at the forefront, there are numerous questions that remain unanswered. For example, the IL-24 receptor expression in various tissues and different pathological conditions remains unknown. The fundamental question of why IL-24 protein expression is lost in cancer cells although mRNA is detectable has not been studied. Since IL-24 protein can exert antitumor activity, what are the limitations in producing large quantities of purified IL-24 protein for testing protein-based therapy? Finally, does post-translational modification (PTM) play a role in the IL-24 protein attaining different functional properties such as tumor suppressor function versus cytokine function and also affect intracellular localization? Last but not the least, till date very few studies have focused on IL-24-based systemic therapy. Majority of the studies and examples cited in this article have used intratumoral therapy and have used adenovirus as a gene delivery vehicle. However, clinical experience indicates cancer is often detected in patients when the disease has metastasized requiring systemic therapy. Thus, it is of great importance and of clinical relevance to develop and test delivery vehicles carrying IL-24 gene or IL-24 protein that can be administered systemically and are safe.

With the advent of nanotechnology, we are not very far from combining nanotechnology platforms for IL-24-based therapy. Thus, systemic IL-24-based nanotherapy will become a reality in the future and become available for clinical testing.

## Competing interests

The authors declare that they have no competing interests.

## Authors’ contributions

JP, AM, and RR – wrote the manuscript; AM and RR – reviewed and edited the manuscript. All authors read and approved the final manuscript.
